# Endoscopic treatment of spinal trauma at the thoracolumbar junction

**DOI:** 10.4103/0019-5413.36987

**Published:** 2007

**Authors:** Rudolf Beisse

**Affiliations:** Spine Center, BG Unfallklinik Murnau, Prof.-Küntscher-Str.8, D-82418 Murnau, Germany

**Keywords:** Anterior decompression, endoscopic spine surgery, spinal trauma, thoracolumbar junction

## Abstract

Attempts of treating unstable fractures of the thoracolumbar junction by posterior reduction and fixation alone often result in a significant loss of correction, especially in lesions where a severe destruction of the vertebral body and the intervertebral disc is present. The conventional open approaches like classic thoraco-phreno-lumbotomy produces additional iatrogenic trauma at the lateral chest and abdominal wall which not rarely leads to intercostal neuralgia, as well as post-thoracotomy syndromes. The endoscopic trans-diaphragmatic approach described below opens up the whole thoracolumbar junction to a minimally invasive procedure allowing one to perform all the procedures needed for a full reconstruction of the anterior column of the spine like corpectomy, decompression, vertebral body replacement and anterior plating. The key to address also the subdiaphragmal and retroperitoneal section of the thoracolumbar junction is a partial detachment of the diaphragm which runs along the attachment at the spine and the ribs. The technique was published first in 1998 and has been used now in 650 endoscopic procedures at the thoracolumbar junction out of a total of more than 1300 thoracoscopic operations of the spine in the BG Unfallklinik Murnau, Germany since 1996.

Dorsal stabilization techniques based on the fixateur interne principle^1^ are regarded as primary treatment for unstable spine injuries. Certain types of fractures associated with extensive destruction or defects of the vertebral body and intervertebral disc require anterior reconstruction of the load-bearing anterior column of the spine to prevent significant loss of correction.^2^ The classic open approach method to the thoracolumbar junction of the spine is the thoraco-phreno-lumbotomy, described first by Hodgson.^3^ It perfectly exposes the thoracolumbar spine after having detached the diaphragm, but it also creates an extensive additional trauma to the lateral chest and abdominal wall including the rib cage. Further development of endoscopic technique and standardization of the operating procedure have now made it possible to perform the operation on the ventral section of the thoracic and thoracolumbar spine using minimally invasive thoracoscopy. Thus complete or partial corpectomy, anterior decompression by resection of the posterior wall of the vertebral body, the replacement of the vertebral body using bone graft or a cage and anterior instrumentation can be performed using the minimally invasive technique described below.

## INDICATION

The anterior thoracoscopic approach is indicated in the following indications (usually in combination with posterior instrumentation):

- Fractures and unstable injuries located at the thoracolumbar junction from T11 to L2.
- Fractures classified as A 1.3, A 2, A 3, B and C according to the AO classification^4^ and unstable injuries with significant curvature disturbance of 20° and more in the sagittal or frontal plane.
- Posttraumatic, degenerative or tumorous narrowing of the spinal canal.
- Discoligamentous segmental instability.
- Posttraumatic deformity (Anterior release and reconstruction)

In fractures of Types B and C posterior instrumentation is mandatory. In other types it is optional. The decision of how many segments have to be fused will be made according to the CT findings. If the lower half of the fractured vertebra is intact, we go for a monosegmental fusion, where the fractured vertebra and the lower disc will be preserved to achieve a shortest possible construct and fusion. In these cases we use a tricortical bone graft for partial vertebral replacement. In all the other cases a bi- or more- segmental fusion and instrumentation is performed using titanium cages filled and surrounded with bone harvested from the corpectomy.

The patient should be informed about the donor site morbidity due to harvesting of the bone graft from the iliac crest, direct or indirect injuries to the greater vessels (aorta, vena cava) and injuries to the heart and/or lungs, spleen, kidney, ureter and thoracic duct. There is a risk of injury to the spinal cord, spinal nerves and sympathetic trunk with neurological deficits (deafferentation syndrome) and sympathetic dystrophy The patient should be sensitized about the possibility of conversion to a conventional “open” thoracotomy. The risk of pseudoarthrosis with instability and significant loss of correction, implant loosening, implant failure, infections at the target area, as well as at the donor site are the same as with any other open procedure. The patient may develop restricted pulmonary function due to fibrosis, scarring, atelectasis or pleural effusion, diaphragmatic hernia,and may need anti-thrombotic medication, and blood transfusion in emergency.

## TECHNICAL REQUIREMENT^5^

**Figure 1:**
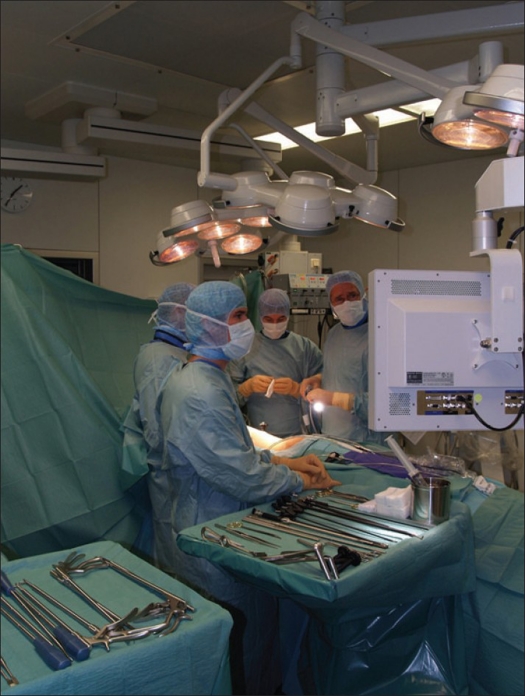
Shows operating room-Setup in thoracoscopic spine surgery

For image transmission we use a rigid 30-degree endoscope connected to a xenon light source and a high-definition three-chip camera unit. The image is transmitted onto three flat screen monitors mounted on a freely positionable endoscopy tower. The tower is also equipped with a suction and irrigation device, a generator for the ultrasonic knife and a digital image and recording system (Storz, Tuttlingen Germany) [Figure 1].

For soft tissue preparation, disc and bone resection specially designed long instruments are used, which are inserted into the chest cavity via black treaded soft trocars with a diameter of 11 mm. The use of black-colored trocars reduces the effect of light reflections. All instruments are offered by several manufacturers as a complete set. For vertebral replacement in bisegmental fusion procedures we use distractable titanium cages, like Synex II^®^ (Synthes) or Hydrolift^®^ (Aesculap, Tuttlingen, Germany). For ventral instrumentation we use the MACS-TL- System^®^ (Modular-Anterior-Construct-System / Thoracolumbar by Aesculap, Tuttlingen, Germany)^6^ since its introduction into the market in 1999, a constraint plate and screw system, which is designed for the endoscopic and open approach technique.

## ENDOSCOPIC APPROACH TO THORACOLUMBAR JUNCTION

**Figure 2:**
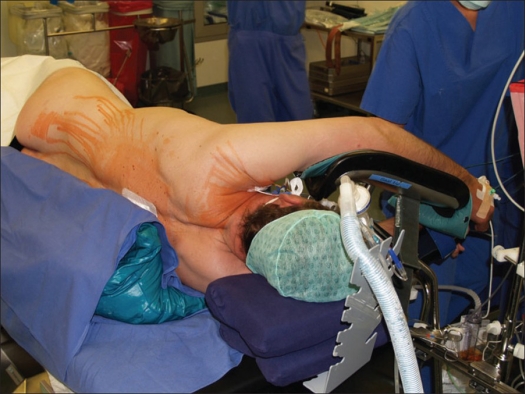
Shows positioning of the patient

**Patient position** [Figure 2] - All operations on the thoracic spine and thoracolumbar junction are performed with the patient lying on his or her side. The side of approach is determined from preoperative computed tomography (CT) scans and depends on the position of the major vessels in the scans and the surgery that is planned. Because of the great variability in vascular anatomy, we no longer set firm rules for selecting the approach side, depending on the height of the lesion. The patient is stabilized in the lateral decubitus position with four supports and a special U-shaped cushion for the legs. Also a vacuum mattress can be used.

**Marking the portals**- Four portals are used: the scope portal, working portal, suction / irrigation portal and retractor portal. Their location is crucial for the whole endoscopic operation and especially the location of the working portal will influence the accuracy and the course of the resection of the vertebra as well as the instrumentation. For this reason, we first display the lesion in the lateral projection (with reference to the patient's body) under precise adjustment of the image intensifier and use a marker to draw the injured spinal section onto the skin of the lateral thoracic wall. The working portal is drawn directly above the lesion. The trocar for the endoscope is marked caudally or cranially to the working portal, depending on the height of the lesion and following the axis of the spine. The distance from the working portal is about two intercostal spaces. The entry points for suction and irrigation and for the retractor are then located ventrally from these portals.

### Approach

**Figure 3:**
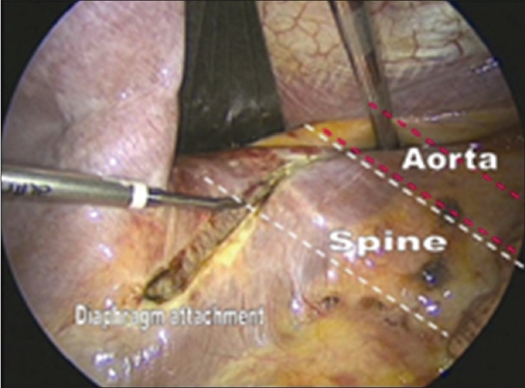
Partial detachment of the diaphragm using an ultrasound knife

**Figure 4:**
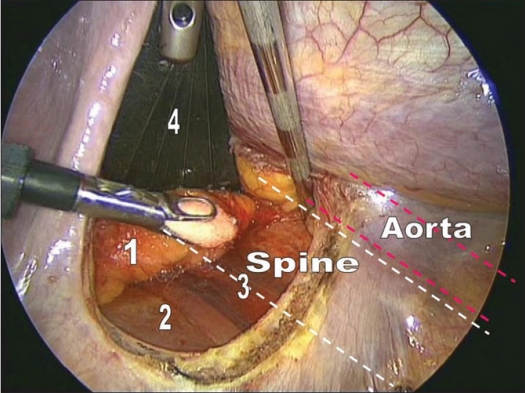
Exposure of subdiaphragmal part of the thoracolumbar junction. The structure shown are - 1. retroperitoneal fat, 2- quadratus lumborum muscle, 3- psoas muscle, 4- fan shaped retractor

The dome-like diaphragm is firmly connected at its margins with the sternum, ribs and spine and arches up into the thoracic cavity.^7^ Topographically, the attachment sites of the diaphragm to the spine are at the level of the first lumbar vertebra. The lowest point of the thoracic cavity projects with the phrenicocostal sinus at the level of the base plate of the second lumbar vertebra. This configuration makes it possible to place a trocar intrathoracically in the phrenicocostal sinus. After incision of the diaphragmatic attachment to the spine, the trocar provides access to the retroperitoneal section of the thoracolumbar junction down to the base plate of the second lumbar vertebra. The incision must be 4 to 5 cm long. Access to the L1-vertebra can be obtained with a shorter incision of 2 to 3 cm. To prevent a postoperative diaphragmatic hernia, we prefer an incision that runs parallel to the diaphragmatic attachment [Figures 3 and 4]. Because of the dome like architecture of the diaphragm, a semicircular incision parallel to the attachment causes the dissected margins to adhere spontaneously under the influence of the intra-abdominal pressure. In contrast, a radial incision aligned with the spine in direct proximity to the orifices of the aorta and esophagus weakens the diaphragm fixation and causes the margins of the incision to gape. We recommend that every incision in the attachment longer than 2 cm be sutured endoscopically to avoid a hernia.

## ANTERIOR DECOMPRESSION AND RECONSTRUCTION

The reconstruction of the anterior spine proceeds in the following standardized steps:

1. K-wires are inserted as landmarks into the adjacent intact vertebral bodies and they also define the entry point for the cannulated screws of the implant which will be inserted subsequently [Figure 5].

**Figure 5:**
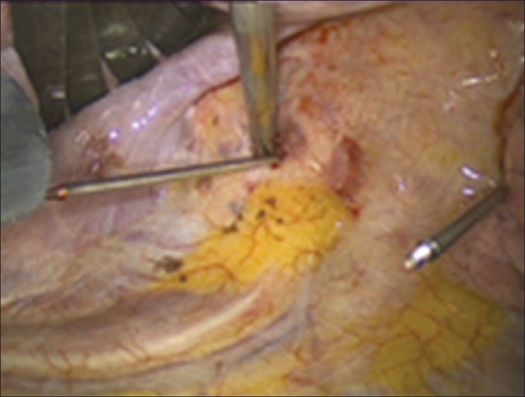
Treatment of a T 11-fracture- insertion of the K-wires in T 10 and T 12
2. The segmental vessels are exposed with an elevator, pulled down and mobilized using a 90°- angled holder, ligated with titanium clips and resected. The lateral vertebral body wall is exposed [Figure 6].

**Figure 6:**
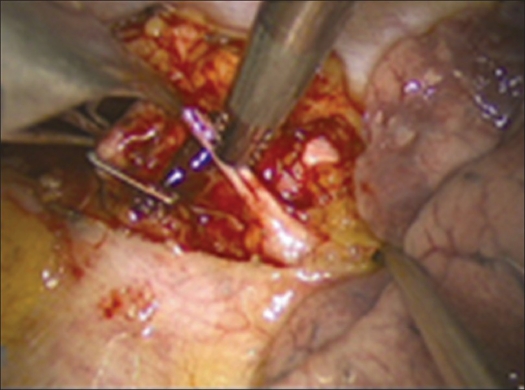
Clipping the segmental vessels
3. The screws are inserted [Figure 7].

**Figure 7:**
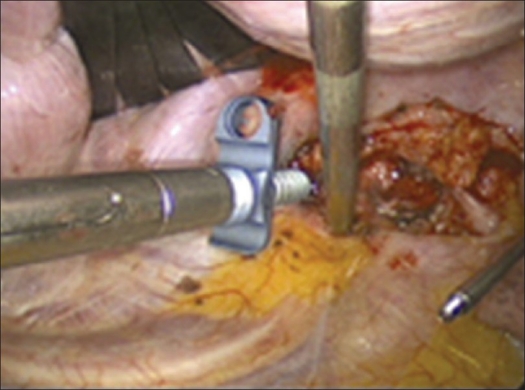
Insertion of the first screw and clamping element into the vertebral body of T12
4. The intervertebral disc is incised. The ventral and dorsal extent of the partial corporectomy is marked using an osteotome. In monosegmental treatment, the caudal limit of the resection is defined using the image intensifier in the anteroposterior projection and osteotomy of the residual vertebral body is performed. The fragments and intervertebral disc material are removed with rongeurs [Figure 8]. Particular importance is placed on removing the disc material and freshening up the endplates of the adjacent vertebrae.

**Figure 8:**
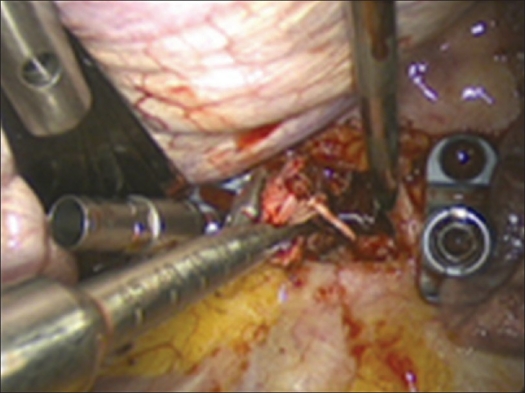
Partial corpectomy of T11 with a rongeur
5. After the length of the defect has been measured a tricortical bone graft or vertebral body replacement device of the corresponding length is implanted [Figure 9].

**Figure 9:**
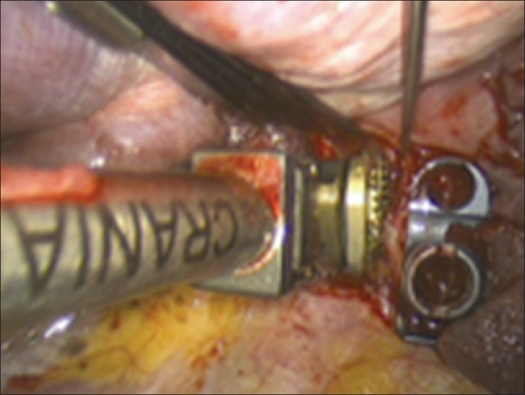
Implantation of the vertebral body replacement device (Synex-Cage II)
6. Building on the K-wires or screws inserted in the first step of the operation, a ventral instrumentation is performed routinely. For this, a constrained plate and screw implant is used as described above to achieve a stable environment that will facilitate the success of the fusion [Figures 10 and 11.]

**Figure 10:**
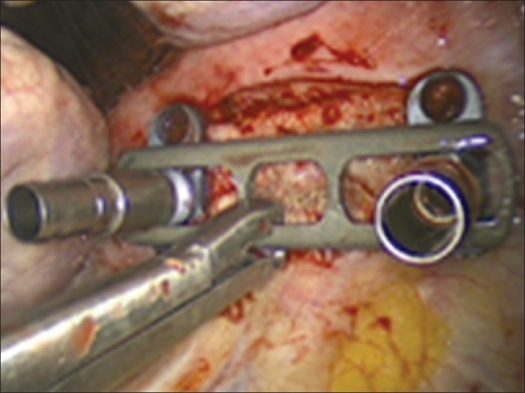
Attachment of the plate

**Figure 11:**
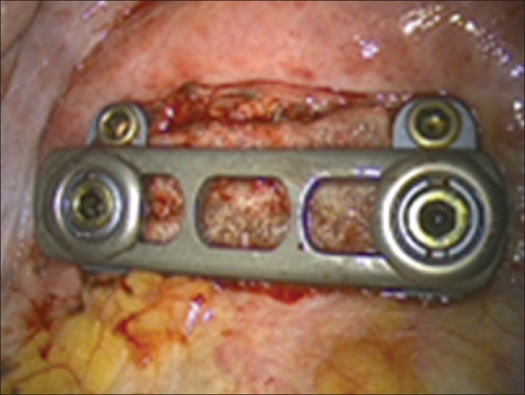
Final result of anterior instrumentation (MACS-TL-constraint plates system)
7. The operation concludes with manual endoscopic suturing of the diaphragm incision and the placement of thoracic drainage. Reinflation of the lung is monitored endoscopically and the four skin incisions are sutured layer by layer after the trocars have been removed.

Anterior decompression^8^ also starts with a partial corpectomy of the injured vertebra which should be perfectly performed before starting with the proper decompression procedure which is recommended to be performed as follows:

1. The key site is mostly the intact pedicle, because the protruded fragment will be trapped in between the two intact pedicles inhibiting the reduction of the fragment. The first step is therefore the exposure of the ipsilateral pedicle. The fibers of the psoas muscle as well as the nerve root is retracted using a nerve root retractor which is inserted via an additional 5 mm incision dorsal to the working portal.
2. The pedicle can be weakened using a high-speed burr and dissected afterwards using a punch.
3. Under direct view to the dura the upper postero-lateral wall of the vertebral body is to be removed and the protruded fragment of the posterior wall can now be pushed away from the dura and removed step by step with a kerrison rongeur.
4. The extent and completeness of the decompression of the whole anterior circumference of the dura down to the contralateral pedicle has to be checked under C-arm control with a nerve hook.
5. The procedure continues with Step 3 of the standard procedure. Instead of tricortical bone graft we recommend using a titanium cage for vertebral body replacement due to its conveyed higher stability and resistance against dislocation.

## RESULTS

Several studies within the series of more than 1300 endoscopic procedures have been performed during the last 11 years, since we introduced the technique in our operative spectrum of spine surgery in 1996. In a collective study by the Berufsgenossenschaftliche Unfallklinik Murnau and Stanford University, Californa,^6^ data on 220 patients with unstable injuries to the thoracolumbar junction in the region of the 12^th^ thoracic and first lumbar vertebra were recorded between May 1996 and June 2002. One hundred and eighty-six patients were from the clientele of the BG-Klinik Murnau and 34 from the Stanford University patient base. The average age was 36. The followup period was between four months and six years, with a mean of two years. A neurological deficit was present in 42% of the patients, categorized as follows according to the Frankel Scale:16% D, 6% C, 5% C and 15% A with complete neurological deficit symptoms. Eighty-nine patients (40.5%) presented with a fracture of the 12^th^ thoracic vertebra and 131 patients (59.5%) had a fracture of the first lumbar vertebra.

The injuries were categorized according to the AO classification. All the patients with B and C injuries were primarily dorsally reduced and stabilized. Thus, of the 220 patients 64.5% (142) were stabilized dorsoventrally and 35.5% (n = 78) of the patients with preoperatively diagnosed Type A compression injuries were exclusively stabilized ventrally. Between 1996 and 1999 we used the Z Plate (Sofamor-Danek) for ventral instrumentation and since November 1999 the constraint plate and screw MACS TL System (Aesculap) have been used for the endoscopic procedure. At this point of time we predominantly used a tricortical bone graft from the iliac crest as a vertebral body replacement (84%) and much less frequently (16%) an extendable titanium cage (Synex^®^, Synthes). At one-year followup complete fusion could be identified in 85% of the exclusively ventrally treated fractures and in 90% of the dorsoventrally treated fractures. Reconstruction of the spine profile was successful in over 90% of the cases.

The operating time at that time was between 70 min and 9 h in our first operations performed, with an average operating time of 3.5 h. The times needed for the retroperitoneal section were between 10 and 20 min including the suture of the diaphragm. With specially developed implants and instruments for endoscopic technique and after a learning curve the mean operating time could be shortened to 90-120 min.

**Figure 12:**
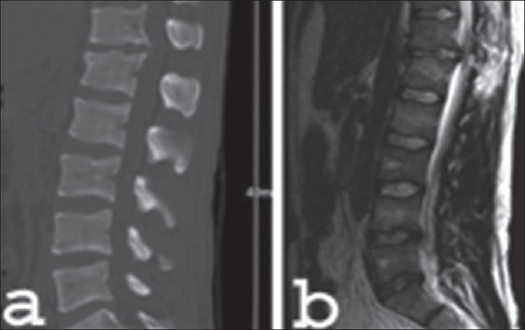
Reconstruction sagittal CT scan (a) and mid sagittal T2WI MRI scan (b) shows flexion destraction type of lesion at D12-L1 with compromise of spinal canal due to disc herniation between T12- L7

**Figure 13:**
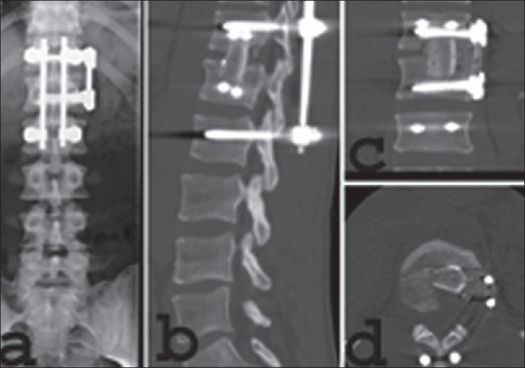
Posterior reduction and fixation followed by thoracoscopic anterior decompression and mono-segmental reconstruction using tricortical bone graft and anterior instrumentation. Plain X-ray AP (a) and lateral (b) coronal reconstruction C.T. (c) and axial CT scan (d) shows insitu posterior implant, adequate anterior decompression and anterior tricortical bone graft with anterior instrumentation

**Figure 14:**
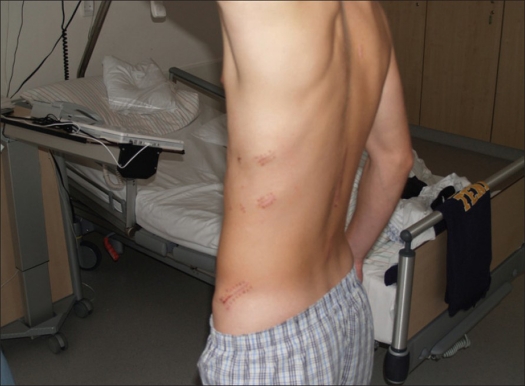
Clinical photograph of the same patients after thoracoscopic reconstruction of the L1-vertebra shows scar

Illustrated case 1: A 22-year-old man fell in a snowboarding accident. He felt immediate severe pain at the thoracolumbar junction as well as numbness in his legs. The CT scan showed a flexion-distraction injury (Type B lesion) at the level of T12/L1 with a severe compromise of the spinal canal due to a deformity and contusion of the posterior wall and a traumatic herniation of the T12/L1 disc [Figure 12]. An ASIA scale D-grade neurological deficit was found. Immediate dorsal reduction and bisegmental fixation was performed using an internal fixator. The partial resection of the posterior wall of T12 and L1, including the disc and the adjacent endplates according to the preoperative planning was performed by anterior thoracoscopic approach. Partial corpectomy of L1 was performed to achieve a monosegmental fusion. A tricortical bone graft was inserted followed by anterior monosegmental instrumentation [Figure 13]. Five months after the operation, the CT scan demonstrated a complete monosegmental fusion. The dorsal fixator was then removed to achieve a monosegmental fusion. The patient had recovered fully from the neurological deficit with minimal scar on thoracic wall [Figure 14].

**Figure 15:**
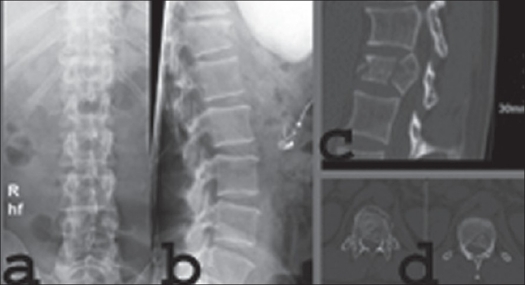
Plain x-ray AP (a) and lateral (b) sagittal reconstructioin CT (c) and axial CT scan (d) Spinal shows burst fracture L1 vertebrae with canal compromise

**Figure 16:**
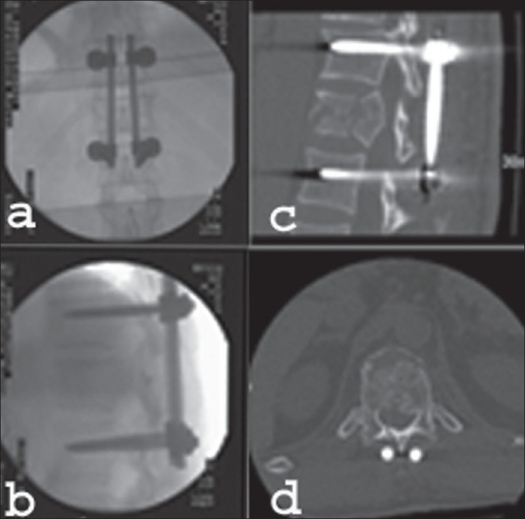
Plain x-ray AP (a) lateral (b) sagittal reconstruction CT scan (c) and axial CT scan (d) shows dorsal reduction and instrumentation of T12-L2 using internal fixator

**Figure 17:**
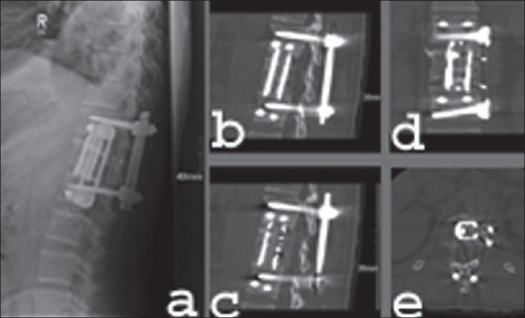
Post operative x-ray lateral view (a) reconstructed sagittal CT scan (b and c), coronal reconstruction (d) and axial CT scan (e) after thoracoscopic anterior decompression of the same patient shows bi-segmental reconstruction with distractable cage filed with cancellous bone and reinforced with ante-rior instrumentation

Illustrated case 2: A 54-year-old woman suffered from L1-fracture in a motorcycle accident. The CT scan showed a complete burst fracture Type A 3.3 according to the AO Clasification^4^ [Figure 15].A one-stage procedure with primary posterior reduction and fixation using an internal fixator (Universal Spine Spine System^®^, Synthes) and secondary anterior reconstruction including vertebral body replacement with a distractable titanium cage (Synex^®^, Synthes) and anterior instrumentation (MACS TL^®^, Aesculap) was performed [Figures 16,17].

### Complications

Given the direct proximity of vital structures, the potential for complications related to surgical operations on the spine must be regarded as high. Compared to the thoracic spine, the risk of neurological complications is less because the spinal cord at the level of the thoracolumbar junction tapers and the spinal canal is relatively wide. However, even more attention must be paid to the ventrally situated vessels. Injury to these vessels represents a life-threatening complication that must be controlled by immediate thoracophenotomy with extensive exposure, mobilization and tying of the vessels.

Leakage of the aorta was recorded twice in the whole series of endoscopic procedures at the thoracolumbar junction since 1996 (two out of 650 cases). In both cases we converted to a thoracotomy and treated the leakage by direct suturing after having mobilized the aorta. We recorded two cases of splenic lesion; of them in one case splenectomy became necessary.

There was no neurological deficit and no diaphragmal hernia. We saw one lesion of the thoracic duct during the anterior release procedure in a case of posttraumatic deformity, which could be identified and closed with staples and fibrin glue.

## DISCUSSION

The thoracolumbar junction of the spine represents at the same time the border region between the thoracic and abdominal cavity and the section of the spine where most of the injuries occur. In our clientele 63% of the patients with a trauma of the truncal spine suffered from an injury at the thoracolumbar junction. Among those the first lumbar vertebra was affected in 44%. The lower limit of the thoracoscopically accessible was generally claimed to be the first lumbar vertrebra,^9^ which was not enough to perform a reconstruction of the first lumbar vertebra using the bisegmental technique from T12 to L2. With partial detachment of the diaphragm running parallel to the diaphragmatic attachment, as described by us and published in 1998,^10^ it is also possible to reach the region of the second lumbar vertebra near the baseplate under thoracoscopic conditions.

As shown by the collective study on 220 patients by the BG Unfallklinik Murnau and Stanford University, California in 2004,^7^ the approach to the thoracolumbar junction described above which was considered as difficult, has a low complication rate. Crucial for successful implementation of this technique is an incision parallel to the attachment of the diaphragm onto the spine and the ribs. The incision should be closed by suturing to avoid a diaphragmatic hernia. The occurrence of a diaphragmatic hernia must be considered as probably the most serious potential complication and this has so far not occurred in any case in which the technique we recommend has been used.

Dickman and Rosenthal^8^ described another approach to the thoracolumbar junction in which the patient is placed in a reverse Trendelenburg position in order to shift the intraabdominal organs in a caudal direction and relax the diaphragm. Separation of the pulmonary ligament and mobilization of the pleura is recommended to give access to the 12^th^ thoracic and first lumbar vertebrae. For regions lying lower regions the authors recommend the use of retroperitoneally positioned portals.

According to our concept of reconstruction of the load-bearing anterior column as a fusion procedure, our aim is to preserve the anterior ligament together with a thin slice of the anterior wall, to resect the central part of the vertebra which will be replaced by a bone graft or cage, to decompress the spinal canal if a neurological deficit in combination with a significant narrowing of the canal i.e. more than 30% is present and to increase the stability and stiffness by a routine anterior instrumentation using a constraint plate and screw system.

The argument for the neccessity of ventral instrumentation in cases where a dorsal stabilization system has already been implanted is still going on. In biomechanical studies, Knop *et al.*^11^,^12^ were able to demonstrate adequate stability using a fixateur interne and an extendable titanium cage (Synex^®^) that is “braced” against the fixateur. Other current biomechanical studies^13^,^14^,^15^ have shown that ventral instrumentation has a substantial reinforcing influence on the primary stability of the spine section treated with this method. However, the crucial question of how much stability a fusion requires for bone ingrowth remains open.

The controversy on the indication for anterior decompression is also not yet concluded. Our concept foresees immediate dorsal reduction and stabilization for indirect reposition of the posterior margin fragment in every case of an unstable injury to the thoracolumbar junction combined with a neurological deficit. In around 80% of the cases with a severe compromise of the spinal canal a sufficient relief of the neural structures could be achieved, which was proven by intraoperative myelography. If there is a block to the flow of contrast medium, dorsal decompression is immediately performed. Endoscopic anterior decompression as an elective operation then serves to remove the ventrally compressing fragments with a view to avoiding longterm consequences.^13^

The endoscopic technique for decompression has the advantage of the excellent view of the fracture site with the 30° scope which we routinely use and which, depending on the rotation of the scope and the nearness to the object, can be compared to the view obtained with the surgical microscope. We analyzed the efficacy of the procedure and published the technique and results in 2005.^6^ The study showed an equivalent effectiveness in spinal canal clearance of approximately 100% compared to the open ventral technique. The average operating times have since been reduced substantially.

## CONCLUSION

In the last 15 years, endoscopic procedures on the spine have become an alternative standardized spine surgery. Through the trans-diaphragmatic approach it has been possible to open up the thoracolumbar junction, including the retroperitoneal segments of the spine, using the endoscopic technique. With the extension of the technique to the retroperitoneal sections of the thoracolumbar junction it was possible at the same time to increase the indication spectrum of the endoscopic technique substantially, so that it today includes complete fracture treatment with vertebral body replacement and ventral instrumentation as well as anterior decompression of the spinal canal. The complication rate of the endoscopic procedure is of the same scale as that known from the open procedures, with clear advantages in terms of the reduced access morbidity associated with the minimally invasive technique.
